# The Case for a Global Ban on Asbestos

**DOI:** 10.1289/ehp.1002285

**Published:** 2010-07-01

**Authors:** Joseph LaDou, Barry Castleman, Arthur Frank, Michael Gochfeld, Morris Greenberg, James Huff, Tushar Kant Joshi, Philip J. Landrigan, Richard Lemen, Jonny Myers, Morando Soffritti, Colin L. Soskolne, Ken Takahashi, Daniel Teitelbaum, Benedetto Terracini, Andrew Watterson

**Affiliations:** 1 Division of Occupational and Environmental Medicine, University of California–San Francisco, San Francisco, California, USA; 2 Environmental Consultant, Garrett Park, Maryland, USA; 3 Department of Environmental and Occupational Health, Drexel University School of Public Health, Philadelphia, Pennsylvania, USA; 4 Department of Environmental and Occupational Medicine, Robert Wood Johnson Medical School, Piscataway/New Brunswick, New Jersey, USA; 5 Former HM Inspector of Factories, London, United Kingdom; 6 Chemical Carcinogenesis, Office of the Director, National Institute of Environmental Health Sciences, National Institutes of Health, Department of Health and Human Services, Research Triangle Park, North Carolina, USA; 7 Center for Occupational and Environmental Health, New Delhi, India; 8 Global Health Program, Mount Sinai School of Medicine, New York, New York, USA; 9 National Institute for Occupational Safety and Health (retired), Canton, Georgia, USA; 10 Center for Occupational and Environmental Health Research, University of Cape Town, Cape Town, South Africa; 11 European Foundation for Oncology and Environmental Sciences, Bologna, Italy; 12 School of Public Health, University of Alberta, Edmonton, Alberta, Canada; 13 Department of Epidemiology, University of Occupational and Environmental Health, Kitakyushu, Japan; 14 School of Public Health, University of Colorado, Denver, Colorado, USA; 15 Center for Cancer Prevention, University of Torino, Torino, Italy; 16 Occupational and Environmental Health Research Group, University of Stirling, Stirling, Scotland

**Keywords:** asbestos, asbestos cancer pandemic, asbestos-related diseases, ban, cancer, chrysotile, controlled use, disinformation, mesothelioma, product defense

## Abstract

**Background:**

All forms of asbestos are now banned in 52 countries. Safer products have replaced many materials that once were made with it. Nonetheless, many countries still use, import, and export asbestos and asbestos-containing products, and in those that have banned other forms of asbestos, the so-called “controlled use” of chrysotile asbestos is often exempted from the ban. In fact, chrysotile has accounted for > 95% of all the asbestos used globally.

**Objective:**

We examined and evaluated the literature used to support the exemption of chrysotile asbestos from the ban and how its exemption reflects the political and economic influence of the asbestos mining and manufacturing industry.

**Discussion:**

All forms of asbestos, including chrysotile, are proven human carcinogens. All forms cause malignant mesothelioma and lung and laryngeal cancers, and may cause ovarian, gastrointestinal, and other cancers. No exposure to asbestos is without risk. Illnesses and deaths from asbestos exposure are entirely preventable.

**Conclusions:**

All countries of the world have an obligation to their citizens to join in the international endeavor to ban the mining, manufacture, and use of all forms of asbestos. An international ban is urgently needed. There is no medical or scientific basis to exempt chrysotile from the worldwide ban of asbestos.

The Collegium Ramazzini first called for a universal ban on the mining, manufacture, and use of asbestos more than a decade ago (Collegium Ramazzini 1999). All forms of asbestos are now banned in 52 countries, and safer products have replaced many materials that once were made with it. Nonetheless, a large number of countries still use, import, and export asbestos and asbestos-containing products. In many countries that have banned other forms of asbestos, the so-called controlled use of chrysotile asbestos is exempted from the ban, an exemption that reflects the political and economic influence of the asbestos mining and manufacturing industry lobbies.

All forms of asbestos cause asbestosis, a progressive, debilitating fibrotic disease of the lungs. All forms of asbestos also cause malignant mesothelioma and lung and laryngeal cancers, and may cause ovarian, gastrointestinal, and other cancers (Straif et al. 2009). More than 20 years ago, asbestos was declared a proven human carcinogen by the U.S. Environmental Protection Agency (U.S. EPA 1986), the International Agency for Research on Cancer (1977) of the World Health Organization (WHO), and the U.S. National Toxicology Program (NTP 1980). The scientific community is in overwhelming agreement that there is no safe level of exposure to asbestos (Welch 2007; Welch et al. 2009). Moreover, there is no evidence of a threshold level below which there is no risk of mesothelioma (Hillerdal 1999).

## The Asbestos Cancer Pandemic

### Occupational exposures to asbestos

About 125 million people around the world are exposed to asbestos in their work environments (WHO 2006), and many millions more workers have been exposed to asbestos in years past. As noted by Stayner et al. (1997), the U.S. National Institute for Occupational Safety and Health (NIOSH) has estimated that current occupational exposures to asbestos, even at the U.S. Occupational Safety and Health Administration (OSHA) permissible exposure limit, will cause five deaths from lung cancer and two deaths from asbestosis in every 1,000 workers exposed for a working lifetime.

In 2000, an estimated 43,000 deaths worldwide resulted from malignant mesothelioma, and a much larger number of lung cancer deaths were due to occupational exposures to asbestos (Driscoll et al. 2005). Population-attributable risk for lung cancer among males exposed to asbestos ranges between 10% and 20% (Albin et al. 1999). An estimated 20,000 asbestos-related lung cancers and 10,000 cases of mesothelioma occur annually across the population of Western Europe, Scandinavia, North America, Japan, and Australia (Tossavainen 2000). The national incidence rates for mesothelioma in Australia are the highest in the world (Leigh and Driscoll 2003).

In the United Kingdom, at least 3,500 people die from asbestos-related illnesses each year, and this number is expected to increase to 5,000 in future years. Asbestos accounts for more than half of the work-related cancer deaths in Great Britain (Rushton et al. 2008). The British mesothelioma death rate is now the highest in the world, with 1,749 deaths in men (1 in 40 of all cancer deaths in men < 80 years of age) and 288 in women in 2005 (Rake et al. 2009). The projected lifetime risk of fatal mesothelioma in all British men born in the 1940s is 0.59%, or about 1 in 170 of all deaths. By 2050, there will have been approximately 90,000 deaths from mesothelioma in Great Britain, 65,000 occurring after 2001 (Hodgson et al. 2005).

### Environmental exposures to asbestos

Nonoccupational, environmental exposure to asbestos from the use of construction materials that contain asbestos is also a serious and often neglected problem throughout the world. In developed countries, large quantities of asbestos remain as a legacy of past construction practices in many thousands of schools, homes, and commercial buildings. In developing countries, where asbestos is used today in large quantities in construction, asbestos-contaminated dust is now accumulating in thousands of communities, with virtually all people burdened with asbestos fibers in their lungs and bodies (Brophy et al. 2007; Kazan-Allen 2005).

Both community-based and industrial exposures to asbestos and asbestiform fibers increase risks for mesothelioma (Pasetto et al. 2005). In a study of women residing in Canadian asbestos-mining communities, Camus et al. (1998) found a 7-fold increase in the mortality rate from pleural cancer. In California, residential proximity to naturally occurring asbestos was significantly associated with increased risk of mesothelioma (Pan et al. 2005); the risk of mesothelioma decreased approximately 6.3% for every 10-km increase in residential distance from the nearest asbestos source. Driece et al. (2009) reported that environmental exposures to asbestos waste on the surfaces of roads and yards in a contaminated community of 130,000 residents in the Netherlands result in several cases of malignant mesothelioma each year. The currently observed increase in female cases of mesothelioma in the United Kingdom, many with no occupational exposure to asbestos, suggests widespread environmental contamination (Rake et al. 2009). In a study in Libby, Montana, (Vinikoor et al. 2010), respiratory symptoms were positively associated with the frequent handling of vermiculite insulation. Residents of this mining community who were children when the mine closed experienced respiratory symptoms associated with asbestos-contaminated vermiculite exposure.

## Science and Controversy

Asbestos is a general term applied to certain fibrous minerals of two configurations: serpentine and amphibole. The only type of asbestos derived from serpentine minerals, chrysotile (also known as white asbestos), accounts for 100% of the asbestos used in the world today (Natural Resources Canada 2006). Amphibole minerals include five asbestos species: amosite, crocidolite, tremolite, anthophyllite, and actinolite. Two of these are the most commercially valuable forms: amosite, or brown asbestos, and crocidolite, or blue asbestos. Other minerals sometimes containing fibers that are not defined by industry as asbestos, such as erionite, taconite, and talc, are clearly capable of causing asbestos diseases, as are certain man-made fibers, including some nanofibers (Dikensoy 2008; Ryman-Rasmussen et al. 2009; Sanchez et al. 2009). The thermal and chemical resistance and tensile strength of asbestos fibers gave rise to a burgeoning industry before their detrimental health effects—which often take years and decades to appear—became known.

The asbestos industry has relied on scientific debates over the roles of fiber types, viruses, and genetics in the development of mesothelioma to obfuscate the problem of asbestos-related disease (Castleman et al. 1998). The risk of lung cancer among workers exposed to chrysotile asbestos increases slightly with exposure to longer and thinner fibers (Loomis et al. 2009). However, efforts to use statistical models to characterize relative cancer potencies for asbestos fiber types and sizes have not been able to overcome limitations of the exposure data. Epidemiologic, experimental, and molecular evidence suggests that the arguments for the role of fiber size relative to dose, dose–response effect, and genetic susceptibility are fraught with enormous uncertainties (Terracini 2007; Tomatis et al. 2007). Scientists from NIOSH (2010) contend that the uncertainties have been so great that these estimates should not be used to determine occupational and environmental health policy until the agency can perform further research. The U.S. EPA has rejected and discontinued work on its proposed methods for quantifying potency factors for partitioned asbestos fiber types and sizes (Silverstein et al. 2009).

Concern has been raised that mesothelioma deaths might be partly attributable to poliovirus vaccines used during the 1950s and 1960s that were contaminated with simian virus 40 (SV40), a monkey virus that is tumorigenic in rodents (Leithner et al. 2006; Price et al. 2007). However, sex- and age-specific trends in pleural mesothelioma incidence rates were not consistent with an effect of exposure to SV40-contaminated poliovirus vaccine. In addition, studies reporting a high prevalence of SV40 DNA in human tumors were based on molecular assays that are prone to false-positive results (Lopez-Rios et al. 2004).

Some researchers have suggested that susceptibility to asbestos-related diseases is related to genetic differences between individuals within populations. A study of a mesothelioma clustering in Turkey advocated the role of genetic susceptibility and familial inheritance in the etiology of the disease (Roushdy-Hammady et al. 2001; Saracci and Simonato 2001). A genetic factor identified in three villages in Cappadocia, Turkey, where 50% of individuals die of mesothelioma, may contribute to the high incidence of the disease. In these villages, genetic predisposition for mesothelioma works together with erionite (Carbone and Rdzanek 2004). However, in European studies the low proportion of familial cases does not suggest the influence of a large genetic component for mesothelioma in blood relatives (Ascoli et al. 2007).

Controversies such as these have helped to make the disease experiences of asbestos-exposed workers and people in asbestos-contaminated communities invisible and uncompensated, allowing the asbestos industry to escape accountability (Braun et al. 2003). The problem extends well beyond asbestos. “Product defense papers” are commissioned by a wide range of industries seeking to blunt regulators’ efforts and to defeat the cases brought by plaintiffs. Even physician-scientists reporting on hazards of asbestos have been disciplined by their politically motivated governments (Joshi et al. 2009).

Industries have the resources to seed the literature with strategic science that is less likely to be subjected to the same scrutiny routinely applied to science that is explicitly case specific (Boden and Ozonoff 2008). Many articles, published primarily in toxicology journals, are termed “product defense” science articles and are frequently sponsored by asbestos interests such as the defendants in personal injury asbestos litigation in the United States (Axelson et al. 2003; Michaels 2008). These articles are distinguished from other science papers in that they are written by scientific consultants and consulting firms that are approached and paid millions of dollars to publish and promote articles used to try to defeat liability claims (Michaels 2006). General Motors, Ford, and Chrysler sponsored the writing of review articles and meta-analyses of previously published work, and paid almost $37 million between 2001 and 2008 to scientist-consultants at ChemRisk and Exponent, Inc., for presentations of these papers at scientific meetings and expert testimony on the articles (Dietz et al. *v*. ACandS Inc. et al. 2009). These companies were defendants in damage suits brought by mechanics over their asbestos exposures and disease arising from automotive friction materials.

When there is consensus in the public health community about the health effects of a compound—particularly one that is as well researched as asbestos—government agencies and other funders are not interested in additional research that will merely demonstrate what is already known. The only people who have an incentive to continue to fund research on the health effects of chrysotile are those with an economic incentive to raise doubt about its harm. Sponsorship by parties involved in litigation leads to an imbalance in the literature (Michaels and Monforton 2007). As a result, subsequent literature reviews that report a predominance of articles reaching a certain conclusion may then mistakenly report there is a new “consensus” in the literature when that consensus is an artifact of sponsorship (Michaels 2009). Wealthy sponsors have simply paid to have more papers published.

A Conference on Asbestos and Mesothelioma was held in May 2010 and was sponsored by both plaintiff and defense lawyers who paid scientists to come to a resort center to discuss asbestos issues (Perrin Conferences 2010). The conference discussed matters on which there is broad scientific consensus that are still questioned as part of the defense in litigation seeking to reject compensation. Such conferences can serve to perpetuate the illusion of uncertainty about issues for which there is ample evidence concerning the dangers of all forms of asbestos. Indeed, asbestos interests have a record of seizing opportunities to challenge the carcinogenicity of chrysotile, trying to create the impression that it is still a matter of legitimate scientific debate; this creates doubt about legitimate scientific findings and renders policy interventions unlikely (McCulloch and Tweedale 2008). The complex ties of the asbestos industry with international groups are numerous and problematic (Ashford et al. 2002; Castleman 2001; LaDou 2004).

## Chrysotile Asbestos

Chrysotile represents nearly 100% of the asbestos produced and used worldwide today (Natural Resources Canada 2006) and 95% of all the asbestos used worldwide since 1900 (Virta 2005). There is general agreement among scientists and physicians, and widespread support from agencies in countries around the world, that chrysotile causes various cancers, including mesothelioma and lung cancer (Agency for Toxic Substances and Disease Registration 2001; American Conference of Governmental Industrial Hygienists 2001; International Labour Organization 2006; International Social Security Association 2004; National Cancer Institute 2003; NTP 2004; OSHA 1994; United Nations Environment Program 1998; WHO 2006; World Trade Organization 2001].

Early suggestions and industry reports that chrysotile might be significantly less dangerous than other forms of asbestos have not been substantiated. Although chrysotile accounts for almost all the asbestos ever used, the asbestos industry continues to claim that asbestos-related cancers are the result of the amphibole varieties (McCulloch 2006). Defenders of the chrysotile asbestos industry contend that “exposure to chrysotile in a pure form seems likely to present a very low if any risk of mesothelioma” (Gibbs and Berry 2008).

The Chrysotile Institute (Montreal, Quebec, Canada), a registered lobby group for the Quebec asbestos mining industry, takes the position that chrysotile can be handled safely (Chrysotile Institute 2008). Numerous epidemiologic studies, case reports, controlled animal experiments, and toxicological studies refute the assertion that chrysotile is safe (Bang et al. 2006; Landrigan et al. 1999; Lemen 2004b; Lin et al. 2007; Smith and Wright 1996; Stayner et al. 1996; Tossavainen 1997). These studies demonstrate that the so-called controlled use of asbestos is a fallacy (Lemen 2004a; Welch et al. 2009). Workers exposed to chrysotile fiber alone have excessive risks of lung cancer and mesothelioma (Frank et al. 1998; Li et al. 2004; Mirabelli et al. 2008).

The Canadian Cancer Society (2010), the Canadian Medical Association (2009), and the Canadian Public Health Association (2010) oppose the export of asbestos to developing countries. The National Public Health Institute of Quebec has published 15 reports, all of them showing a failure to achieve “controlled use” of asbestos in Quebec itself (Takaro et al. 2010). Pat Martin, a member of Canada’s parliament and a former asbestos miner, asks, “If we in the developed world haven’t found a way to handle chrysotile safely, how can we expect them to do so in developing nations?” (Burki 2010).

Some countries have banned forms of asbestos no longer in use anywhere, yet they exempt the use of chrysotile. This exemption reflects the close relationship the asbestos industry has with many governments, the lack of public health information and regulation in these countries, and the lack of compensation for asbestos victims (Castleman and Joshi 2007; Greenberg 2005; Kazan-Allen 2003). The toll in most countries still using large amounts of asbestos may never be fully ascertained or recorded.

## Current Production and Use of Asbestos

Despite all that is known about the dangerous and adverse health effects of asbestos, annual world production remains at > 2 million tons [U.S. Geological Survey (USGS) 2009]. Russia is now the leading producer of asbestos worldwide, followed by China, Kazakhstan, Brazil, Canada, Zimbabwe, and Colombia. These six countries accounted for 96% of the world production of asbestos in 2007. Russia has mines rich enough in asbestos deposits to last for > 100 years at current levels of production (Encyclopedia of the Nations 2010). Most of the 925,000 tons of asbestos extracted annually in Russia is exported.

All forms of asbestos are now banned in 52 countries (International Ban Asbestos Secretariat 2010), including all European Union member countries. Nonetheless, these 52 countries make up less than one-third of WHO member countries. A much larger number of WHO member countries still use, import, and export asbestos and asbestos-containing products (WHO 2006). These are almost all countries in Asia, Eastern Europe, Latin America, and Africa. Most of the world’s people still live in countries where asbestos use continues, usually with few safeguards. More than 85% of the world production of asbestos is used today to manufacture products in Asia and Eastern Europe (Virta 2005). In developing countries, where too often there exists little or no protection of workers and communities, the asbestos cancer pandemic may be the most devastating. China is by far the largest consumer of asbestos in the world today, followed by Russia, India, Kazakhstan, Brazil, Indonesia, Thailand, Vietnam, and Ukraine (United Nations Statistics Division 2009; USGS 2009).

## Position of International Agencies on Asbestos

International organizations have condemned the continuing use of chrysotile asbestos. In 2006, the WHO called for the elimination of diseases associated with asbestos. The WHO supports individual countries in developing national plans to ban asbestos and eliminate asbestos-related disease, stating that “the most efficient way to eliminate asbestos-related disease is to stop using all types of asbestos” (WHO 2007). The International Labour Organization (2006) expressed concern about an evolving epidemic of asbestos-related diseases and passed a resolution to promote a worldwide asbestos ban. The World Trade Organization has accepted the conclusion that the “controlled use” of asbestos is a fallacy (Castleman 2002).

The Rotterdam Convention (2005) is an international agreement intended to regulate global trade in dangerous chemicals—chemicals that have been banned or severely restricted because of their hazards to human health or the environment. It was entered into force in 2004, and 131 nations are currently Parties to the Convention. The goal is to protect the world’s most vulnerable countries—developing countries and countries with economies in transition—against importation of hazardous pesticides and other listed chemicals without their prior informed consent (PIC).

PIC is the core principle of the Rotterdam Convention. This legally binding procedure requires that governments in all countries be provided full information about the risks to health and the environment of each of the hazardous materials regulated by the Convention before importation. Annex III of the Rotterdam Convention lists the chemicals—40 in number—currently covered by the Convention’s PIC requirement: 25 pesticides, 4 severely hazardous pesticide formulations, and 11 industrial chemicals.

Repeated efforts to include chrysotile asbestos under the Rotterdam Convention have failed, not because its Chemical Review Committee has not recommended the listing of chrysotile, but because of the Convention’s requirement for unanimity and as a result of the determined opposition of asbestos mining and manufacturing countries. At the 2008 conference of parties on the Convention, Kazakhstan, Kyrgyzstan, Vietnam, Russia, and Zimbabwe opposed listing chrysotile asbestos in Annex III [IISD (International Institute for Sustainable Development) Reporting Services 2008]. A few asbestos-importing countries thwarted the will of > 100 other countries.

## The Need for a Universal Ban on Asbestos

The profound tragedy of the asbestos pandemic is that all illnesses and deaths related to asbestos are preventable. Safer substitutes for asbestos exist, and they have been introduced successfully in many nations. Currently, asbestos cement products account for > 85% of world consumption (Virta 2005), and in about 100 countries, asbestos-containing pipes and sheets are manufactured to be used as low-cost building materials (Tossavainen 2004). However, these asbestos cement water-pipe products could be replaced with ductile iron pipe, high-density polyethylene pipe, and metal-wire–reinforced concrete pipe. Many substitutes exist for roofing as well as interior building walls and ceilings, including fiber-cement flat and corrugated sheet products that are made with polyvinyl alcohol fibers and cellulose fibers. Virtually all of the polymeric and cellulose fibers used instead of asbestos in fiber-cement sheets are > 10 μm in diameter and therefore nonrespirable (WHO 2005). For roofing, lightweight concrete tiles can be made and used in the most remote locations using locally available plant fibers, such as jute, hemp, sisal, palm nut, coconut coir, and wood pulp. Galvanized iron roofing and clay tiles are among the other alternative materials (World Bank Group 2009).

If global use of asbestos were to cease today, a decrease in the incidence of asbestos-related diseases would become evident in approximately 20 years (WHO 2006). The asbestos cancer pandemic may take as many as 10 million lives before asbestos is banned worldwide and all exposure is brought to an end (LaDou 2004). But the world’s current production of asbestos continues at an alarming rate; therefore; these figures may not reflect the true burden of this pandemic.

An international ban on the mining and use of asbestos is urgently needed. The risks of exposure to asbestos cannot be controlled by technology or by regulation of work practices. Scientists, physicians, and responsible authorities in countries allowing the use of asbestos should have no illusion that “controlled use” of chrysotile asbestos is an effective alternative to a ban on all use of asbestos (Castleman 2003; Egilman and Roberts 2004). Even the best systems of workplace controls cannot prevent occupational and environmental exposures to products in use, or exposures to asbestos discarded as waste. Safer substitute products are in use in countries all over the world where asbestos is banned.

To protect the health of all—now and in future environments—the Collegium Ramazzini again calls on all countries of the world to join in the international endeavor to ban the mining, manufacture, and use of all forms of asbestos.
